# Effect of Thirst-Driven Fluid Intake on 1 H Cycling Time-Trial Performance in Trained Endurance Athletes

**DOI:** 10.3390/sports7100223

**Published:** 2019-10-14

**Authors:** Maxime Perreault-Briere, Jeff Beliveau, David Jeker, Thomas A. Deshayes, Ana Duran, Eric D. B. Goulet

**Affiliations:** 1 Faculty of Physical Activity Sciences, University of Sherbrooke, Sherbrooke, QC J1K 2R1, Canada; Maxime.Perreault-Briere@USherbrooke.ca (M.P.-B.); Jeff.Beliveau@USherbrooke.ca (J.B.); David.Jeker@USherbrooke.ca (D.J.); Thomas.Deshayes@USherbrooke.ca (T.A.D.); 2 Research Centre on Aging, University of Sherbrooke, Sherbrooke, QC J1H 4C4, Canada; 3 Facultad de Organización Deportiva, Universidad Autónoma de Nuevo León, San Nicolás de los Garza, NL 66455, Mexico; ana_duran92@hotmail.com

**Keywords:** cycling, dehydration, endurance performance, fluid balance, thirst

## Abstract

A meta-analysis demonstrated that programmed fluid intake (PFI) aimed at fully replacing sweat losses during a 1 h high-intensity cycling exercise impairs performance compared with no fluid intake (NFI). It was reported that thirst-driven fluid intake (TDFI) may optimize cycling performance, compared with when fluid is consumed more than thirst dictates. However, how TDFI, compared with PFI and NFI, impacts performance during a 1 h cycling time-trial performance remains unknown. The aim of this study was to compare the effect of NFI, TDFI and PFI on 1 h cycling time-trial performance. Using a randomized, crossover and counterbalanced protocol, 9 (7 males and 2 females) trained endurance athletes (30 ± 9 years; Peak $\overset{\cdot }{V}$O_2_∶ 59 ± 8 mL·kg^−1^·min^−1^) completed three 1 h cycling time-trials (30 °C, 50% RH) with either NFI, TDFI or PFI designed to maintain body mass (BM) at ~0.5% of pre-exercise BM. Body mass loss reached 2.9 ± 0.4, 2.2 ± 0.3 and 0.6 ± 0.2% with NFI, TDFI and PFI, respectively. Heart rate, rectal and mean skin temperatures and ratings of perceived exertion and of abdominal discomfort diverged marginally among trials. Mean distance completed (NFI: 35.6 ± 1.9 km; TDFI: 35.8 ± 2.0; PFI: 35.7 ± 2.0) and, hence, average power output maintained during the time-trials did not significantly differ among trials, and the impact of both PFI and TDFI vs. NFI was deemed trivial or unclear. These findings indicate that neither PFI nor TDFI are likely to offer any advantage over NFI during a 1 h cycling time-trial.

## 1. Introduction

Exercise-induced dehydration may impair endurance performance through increased core temperature and perceived exertion and decreased venous return and stroke volume [1]. Adequate fluid replacement during exercise improves cardiovascular and thermoregulatory functions and reduces perceived exertion [2].

Several studies have examined the impact of programed fluid intake (PFI) vs. no fluid intake (NFI) on 1 h high-intensity cycling performance, with most showing that both strategies similarly impact performance [3,4,5,6], while some others showed that PFI either decreases [7] or improves [8] performance, compared with NFI. However, a recently published meta-analysis has concluded that PFI impairs performance during a 1 h high-intensity cycling exercise [9] compared with NFI. Fluid integration into the body during a high-intensity exercise is limited to ~1000 mL·h^−1^ [10]. Thus, in those studies included in the meta-analysis by Holland et al. [9], a large amount of water likely accumulated in the stomach and intestine, owing to the high sweat production that needs to be replaced during high-intensity exercise. Buildup of fluid in those regions can cause abdominal discomfort or pain [4,6,7,11,12,13] and lead to a decrease in endurance performance [7,11,13]. Moreover, as this fluid is not integrated into the body, it cannot fully participate to cardiovascular and thermoregulatory functions. Therefore, it is impossible to conclude without doubt that fluid deprivation is beneficial for a 1 h high-intensity cycling exercise.

With this in mind, it is reasonable to believe that athletes undergoing a 1 h cycling performance in the field would not decide to drink any fluid during exercise, as suggested by Holland et al.’s [9] findings, or, on the contrary, drink sufficient fluid to replace all sweat and urine losses. Goulet and Hoffman [14] recently reported that ad libitum fluid intake optimizes running and cycling performances during exercise lasting 1–2 h, compared with when fluid is consumed in excess of ad libitum drinking. This observation suggests that the optimal regulation of plasma osmolality, not total body water, is required to optimize performance, as suggested by Noakes [15]. However, no studies have directly attempted to determine whether thirst-driven fluid intake (TDFI) may offer any significant advantage over NFI during a 1 h high-intensity cycling performance. Given that TDFI generally leads to significantly lower fluid consumption than PFI [12,16], and presumably to an improved regulation of plasma osmolality, this strategy could potentially prove better than NFI during a 1 h high-intensity cycling exercise which, from a practical point of view, would be reassuring for endurance athletes relying on thirst to replace fluid during such exercise.

Thus, the aim of this study was to compare the effect of NFI, TDFI and PFI aimed at maintaining body mass (BM) loss at ~0.5% on 1 h cycling time-trial performance in endurance-trained athletes. We surmised that the total mean distance completed or mean power output maintained during the time-trial would be higher with TDFI than either PFI or NFI. It was further hypothesized that heart rate, rectal temperature and the perception of thirst and exertion would all be higher in the NFI condition, compared with TDFI and PFI.

## 2. Materials and Methods

### 2.1. Participants

Nine (7 males and 2 females) heat- or partially heat-acclimatized, healthy, endurance-trained competitive cyclists and triathletes participated in this study. Women were tested during the follicular phase (period ranging from the first day of menstruation + the following 13 days) of their menstrual cycle. After explaining the procedures and risks of the study, which were approved by the the CIUSSS Estrie-CHUS Ethics Committee (#2017-1715), written informed consent was obtained from all participants. The specific goals and hypotheses tested during the study were not told to participants to avoid any change in behavior and the placebo or nocebo effect. 

### 2.2. Preliminary Testing and Pre-Experimental Procedures

During the preliminary visit, participants’ resting blood pressure, resting heart rate, BM, height, maximal heart rate, peak oxygen consumption (${\overset{\cdot }{V}O}_{2peak}$) and body composition were measured. Resting heart rate and blood pressure were measured with a digital sphygmomanometer (Welch Allyn 420 series, Skaneateles Falls, NY, USA), nude post-void BM to the nearest 20 g with a digital scale (BX-300 +, Atron Systems, West Caldwell, NJ, USA), height with a wall stadiometer and fat mass and fat-free mass using dual-energy X-ray absorptiometry (Lunar Prodigy, GE Healthcare, Chicago, IL, USA). Peak oxygen consumption was measured on an ergocycle (Ergoselect 100, Ergoline GmbH, Bitz, Germany) using an expired gas analysis system (Cosmed Quark CPET, Cosmed, Chicago, IL, USA) that had been calibrated with gases of known concentration. Three to ten days following the preliminary visit, participants returned to the laboratory for a familiarization trial, which was conducted to (1) familiarize participants with the procedures and measurement techniques used during the experiments, (2) minimize the learning effect, and (3) estimate participants’ sweat rate. Procedures used during the familiarization trial were identical to those used during the experiments. Participants were asked to cover as much distance as possible for a period of 1 h during which they could drink 4 °C water based on thirst sensation. Measurement of sweat rate was necessary to estimate the amount of water intake required for the PFI condition.

Throughout the entire study period, participants were advised to maintain their training routine, except for the last 24 h prior to the familiarization trial where a choice to either rest, train ≤ 45 min at a low to moderate intensity or perform their typical pre-race training was given. Then, the chosen option was replicated over the last 24 h prior to the 3 experimental trials. Also, during the last 24 h prior to the familiarization trial, participants kept and filled a fluid and diet log repeated before the 3 experimental trials. Both lower limb resistance training and dietary supplement intake were forbidden for 48 h and 72 h respectively prior to the familiarization trial as well as the 3 experimental trials. Participants were required to consume 250 mL of water 1 h before the bedtime, which was required to be at the same hour prior to the familiarization trial and all 3 experimental trials. Ninety minutes before reporting to the laboratory for the experiments, participants drank a 220 kcal (carbohydrate: 32 g, protein: 9 g, fat: 6 g, sodium: 200 mg), 242 mL nutritional drink (Kirkland, QC, Canada) to ensure a similar nutritional, hormonal (insulin and glucagon levels) and hydration state prior to the familiarization and all 3 experimental trials. Thirty minutes later, participants consumed 250 mL of water and then after remained fasted.

### 2.3. Experimental Protocol

**Figure 1:**
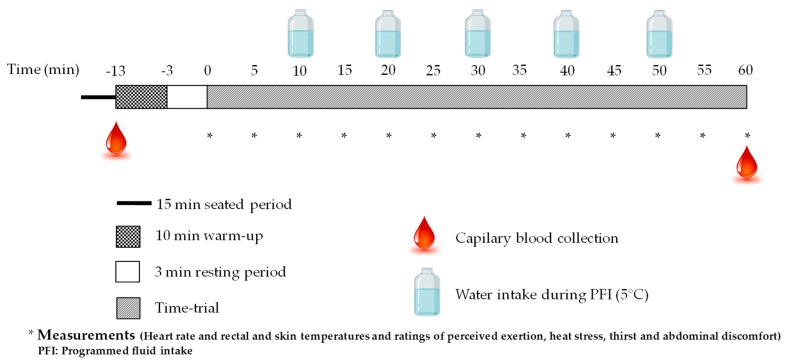
Schematic of the research protocol.

A schematic of the research protocol is found in Figure 1. Following participants’ arrival at the laboratory, all clothes and equipment to be worn during the subsequent exercise period were weighed. Participants voided their bladder, collected a midstream urine sample, were weighed, put on their cycling clothes and shoes, inserted a rectal temperature probe and were instrumented with a Garmin^TM^ chest electrode and four skin temperature probes. Then participants sat quietly for 15 min, after which a capillary blood puncture was taken. Following this procedure, participants entered the environmental chamber and took position on their bike in preparation for the time-trial.

The 1 h cycling time-trial was conducted in an environmental chamber maintained at 30 °C, 50% relative humidity with 800 W·m^2^ of radiance (Apogee SP-110-SS, Apogee, Logan, UT, USA). Convective cooling was provided by three high-speed fans placed in front of the participants, which blew air to the entire legs and torso at 25–30 km·h^−1^. Participants were asked to cover the greatest distance possible during 1 h on a simulated flat course using a Computrainer^TM^ (Racermate, Seattle, WA, USA). They were granted access to power output, speed, distance completed, and elapsed time. Participants use their own bike, ensuring that the same bike was always used throughout the study period and that components and body position were not modified between experimental trials. Prior to commencing the time-trial, participants underwent a standardized 10 min warm-up period. Then, participants rest quietly on their bike for 3 min after which time the time-trial started. 

Throughout the NFI trial participants were completely deprived of water, while during the TDFI trial participants drank water based on their perception of thirst. During the PFI trial, participants drank in a pattern allowing them to finish the time-trial with a BM loss of ~0.5%. The amount of fluid required to maintain BM loss at ~0.5% was estimated using the following formula:

Sweat production measured during the familiarization trial (kg) − (pre-exercise BM (kg)·0.005)(1)

where BM is body mass

Water was provided with an opaque, insulated and tightly sealed bike water bottle. Cold water was provided since it has been demonstrated to facilitate fluid intake [17], reduce core temperature [18] and improve endurance capacity [17,18]. Immediately following the time-trial, a second capillary blood puncture was taken with participants on their bike. Then they stepped off their bike, voided their bladder, collected a midstream urine sample, dried themselves with a towel and were weighed with all equipment and clothes on. 

### 2.4. Measurements

#### 2.4.1. Heart rate, Rectal, Skin and Mean Skin Temperatures

Heart rate was measured continuously using a Garmin^TM^ chest electrode (Garmin, Olathe, KS, USA), rectal temperature with a calibrated YSI 401 rectal probe (Yellow Springs Instrument, Yellow Springs, OH, USA) inserted 15 cm beyond the anal sphincter and skin temperature with calibrated YSI 409 B probes (Yellow Springs Instrument, Yellow Springs, OH, USA) placed on the right side of the body at the forearm, chest, thigh and calf level. The skin probes were held in place with Hypafix dressing (BSN medical, Hamburg, Germany) and mean skin temperature was measured according to Ramanathan [19]. The rectal and skin probes were connected to a high precision digital thermometer (Traceable 4005, Control Company, Webster, TX, USA) and a USB-TEMP data acquisition box (MC measurement computing, Norton, MA, USA), respectively. 

#### 2.4.2. Hemoglobin, Hematocrit and Changes in Plasma Volume and Sodium

Capillary blood punctures were taken from the index of the non-dominant hand at the level of the side of the fingertip perpendicular to the lines of the fingerprint [20] using high blood flow lancing devices (Unistik 3 Dual, Owen Mumford, Marietta, GA, USA). First, the finger was cleaned and disinfected with 70% isopropyl alcohol. After the finger had been pricked, the first blood drop was removed and ~20 µL of capillary blood was collected and distributed in 2 Hemo Point H2 Microcuvettes (Alere, Lowell, MA, USA). Then, another 150 µL of blood was collected in a heparinized microtube and centrifuged for 10 min. Hemoglobin was analyzed by spectrophotometry (Alere H2 Hemopoint, Alere, Lowell, MA, USA). Hematocrit was estimated with the following formula [21]:

F·hemoglobin (g·dL^−1^) (2)

where F is 2.94.

Sodium was measured from plasma sample using ion chromatography (883 Basic IC Plus, Metrohm, Herisau, Swiss). Changes in plasma volume were measured according to Dill and Costill, using the following equation [22]:

100·((Hb_b_/Hb_a_)·((1-Hct_a_/100)/(1-Hct_b_/100))) − 100 (3)

where Hb_b_ is hemoglobin before, Hb_a_ is hemoglobin after, Hct_b_ is hematocrit before and Hct_a_ is hematocrit after.

#### 2.4.3. Sweat Loss, Body Mass Loss, Rate of % Body Mass Loss and Urine Specific Gravity

Sweat loss was taken as the difference in BM from the pre- to post-exercise period, corrected for urine loss and fluid intake during exercise. Loss of mass associated with respiratory water loss and the respiratory exchange of O_2_ and CO_2_ during exercise were not considered and assumed to be similar among trials. Percent BM loss was taken as the difference in BM from the pre- to post-exercise period, relative to pre-exercise BM. The rate of % BM loss was computed by dividing the end-of-exercise % BM loss by exercise time, i.e., 60 min. Urine specific gravity was measured using a digital refractometer (PAL-10S, Atago, Bellevue, WA, USA).

### 2.5. Statistical Analyses

Shapiro-Wilk tests were used to analyze data normality. When normality was respected, one- or two-way repeated measures analyses of variance (ANOVA) were used. Greenhouse-Geisser corrections were applied when sphericity was violated. Abnormally distributed data were tested with Friedman’s ANOVA analyses. Alpha values were corrected with the false discovery rate procedure when multiple pairwise comparisons were performed. Statistical significance was fixed at *p* ≤ 0.05. Based on an estimated coefficient of variation (CV) of 1.17% [23,24], a power analysis (α = 0.05, β = 0.2) revealed that 7 subjects would provide sufficient power to detect a 1.8% (1.5% × CV) change in the distance completed among conditions. The region of practical equivalence (ROPE) [25] and the second-generation *p*-values techniques [26] were used to assess the practical significance of the changes in performance among conditions, based on a smallest worthwhile change in time-trial distance or speed of 0.5 × 1.17% [27]. Moreover, Cohens’s d_z_ effect sizes associated with the changes in distance completed among conditions were computed according to the following formula [28]:

(4) $$t/\sqrt{n}$$

where *t* is the *t*-value of the paired-samples *t*-test and *n* the number of participants.

An effect size <0.20 was considered to be trivial and unsubstantial, between 0.21 and 0.49 small but substantial, between 0.50 and 0.79 moderate and >0.80 large and substantial [29]. All data are reported as means ± standard deviations (SD). 

## 3. Results

### 3.1. Participants and Laboratory Temperature and Relative Humidity

Table 1 shows the physical characteristics of participants. The average ambient temperature and relative humidity inside the laboratory were respectively of 30.0 ± 0.1 °C and 49.2 ± 1.2%, without difference between conditions (both *p* > 0.05).

### 3.2. Hydration State of Participants Prior to the Time-Trials

Table 2 shows data related to the hydration state of participants before each time-trial. Altogether, they indicate that participants were well and similarly hydrated prior to commencing each time-trial. Moreover, thirst perception did not differ (*p* = 0.07) among conditions prior to the beginning of the time-trials (data not shown). 

### 3.3. Fluid Balance

Table 3 reports fluid balance data associated with each cycling time-trial. Participants were successfully hydrated during the PFI condition, since mean post-exercise BM loss amounted to 0.6 ± 0.2% (range 0.32 to 1.05%), which agrees relatively well with our targeted level of 0.5%. Whether reported in absolute terms or relative to BM or distance completed, the amount of fluid consumed with PFI was nearly threefold higher than that when participants drank according to their thirst perception. Although the amount of water consumed was substantially different among trials, total sweat loss, total urine production and urine specific gravity measured following exercise showed no significant difference among conditions. 

### 3.4. Physiological Responses during the Time-Trials

**Figure 2:**
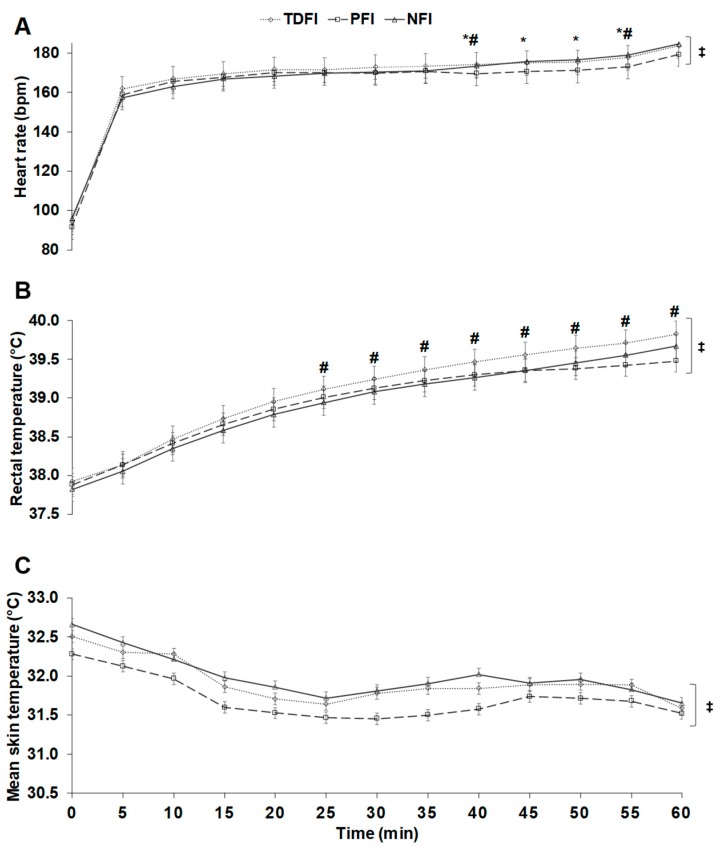
Changes in heart rate (**A**), rectal temperature (**B**), and mean skin temperature (**C**) across time among conditions. Results are means ± SD. * *p* < 0.05 between PFI and NFI, # *p* < 0.05 between PFI and TDFI, ‡ significant time effect.

Figure 2A reports the changes in heart rate over time during each of the three time-trials. A time (*p* < 0.01), interaction (*p* = 0.01) but no condition (*p* = 0.46) effects were observed. The mean heart rates maintained during the time-trials were 171 ± 7, 173 ± 5 and 170 ± 4 beats·min^−1^, representing a mean exercise intensity of 89 ± 5, 90 ± 4 and 88 ± 6% of maximal heart rate for the NFI, TDFI and PFI trials, respectively. Post-hoc comparisons revealed that heart rate was lower with PFI than with TDFI at min 40 (*p* = 0.03) and 55 (*p* = 0.01), and at min 40 (*p* = 0.02), 45 (*p* = 0.04), 50 (*p* = 0.03) and 55 (*p* = 0.04), when compared to NFI while no difference was detected between TDFI and NFI. Rectal (Figure 2B) and mean skin temperatures (Figure 2C) respectively increased and decreased over time (both *p* < 0.01), but no significant difference was observed among conditions for rectal (*p* = 0.08) and mean skin (*p* = 0.49) temperatures. An interaction effect was however observed regarding rectal (*p* = 0.02) but not mean skin (*p* = 0.84) temperature. Post-hoc comparisons showed that only PFI and TDFI were different from 25 min onwards for rectal temperature. The pre- to post-exercise changes in plasma volume were not different among conditions (NFI: −16.3 ± 3.6; TDFI: −16.0 ± 7.7; PFI: −13.3 ± 8.4%, *p* = 0.52), which was also the case regarding the changes in plasma sodium concentration among conditions (NFI: +2.9 ± 8.9; TDFI: +7.0 ± 11.4; PFI: +3.1 ± 12.6 mmol·L^−1^, *p* = 0.44). 

### 3.5. Perceptual Responses during the Time-Trials

**Figure 3:**
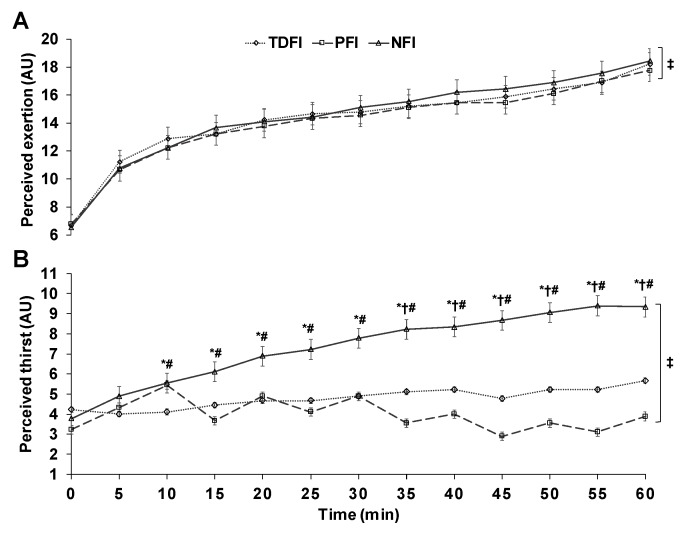
Changes in perceived exertion (**A**) and thirst (**B**) across time among conditions. Results are means ± SD. AU: arbitrary units. * *p* < 0.05 between PFI and NFI, # *p* < 0.05 between PFI and TDFI, † *p* < 0.05 between TDFI and NFI, ‡ significant time effect.

As Figure 3A shows, perceived exertion increased slowly and continuously over time (*p* < 0.01), with no condition (*p* = 0.22) or interaction effect (*p* = 0.17). A significant time, condition and interaction effect was observed among conditions with respect to perceived thirst (Figure 3B) and abdominal discomfort (results not shown) (all *p* < 0.02). Post-hoc comparisons revealed that perceived thirst associated with NFI was significantly higher than either PFI or TDFI from 10 min onwards. Average thirst sensations with NFI, TDFI and PFI were respectively 7.3 ± 2.2, 4.8 ± 1.4 and 4.0 ± 1.6 AU (all *p* < 0.05). Abdominal discomfort was only significantly different between PFI and TDFI at min 50 and 55, and between PFI and NFI at min 55. Average abdominal discomforts with NFI, TDFI and PFI were, respectively, 1.2 ± 0.5, 1.3 ± 0.5 and 1.6 ± 0.9 AU (all *p* > 0.05).

### 3.6. Time-Trial Performance

**Figure 4:**
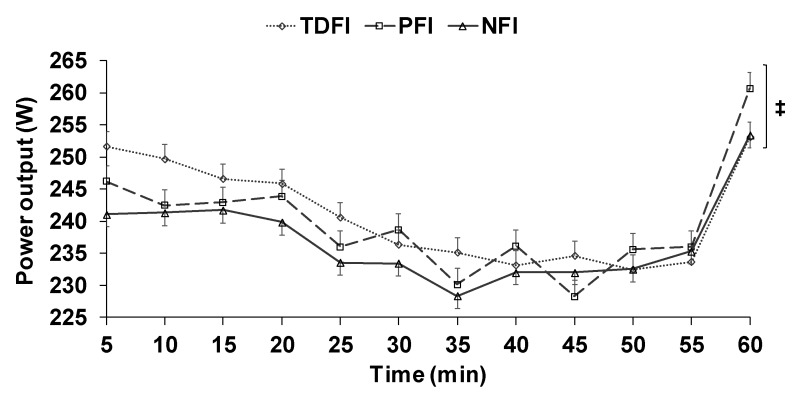
Changes in power output across time among conditions. Results are means ± SD. ‡ significant time effect.

Participants completed the 1 h time-trial at an average speed of 35.6 ± 1.9, 35.8 ± 2.0 and 35.7 ± 2.0 km·h^−1^ for the NFI, TDFI and PFI trials, respectively (*p* = 0.22). The effect sizes associated with the differences on average speed or mean distance completed between NFI vs. TDFI and PFI were both trivial and unsubstantial. Figure 4 shows the changes in power output over time among conditions. A time (*p* < 0.01), but no condition (*p* = 0.25) or interaction (*p* = 0.34) effects were observed. No difference was observed among conditions for the mean power output maintained throughout the time-trials (NFI: 237 ± 31; TDFI: 241 ± 33; PFI: 240 ± 34 W, *p* = 0.23).

Based on the relationships between the highest density interval and the region of practical equivalence (ROPE), as well as based on the second generation p-values-derived observations, from a practical point of view the magnitude of the differences between TDFI and NFI or PFI and NFI is unclear to draw any conclusion about how these strategies impact time-trial performance, compared with NFI. 

## 4. Discussion

This is the first study to compare the effect of NFI, TDFI and PFI during a 1 h cycling time-trial performance in endurance-trained athletes. A meta-analysis [9] had recently concluded that, compared with NFI, a 1 h high-intensity cycling performance was likely to be impaired if all fluid losses through sweat and urine were replaced during exercise. However, it was unknown whether a much lower fluid consumption achieved through the drinking of fluid according to thirst sensation [30] would lead to the same conclusion. From a statistical point of view, our results indicate that there was no difference in the magnitude of the distance completed among the three drinking strategies during the time-trial, and from a practical perspective, that the effects of TDFI and PFI, in comparison to NFI, were trivial and not clear enough to draw any conclusions about how these drinking strategies impact 1 h cycling time-trial performance. The present findings contribute to the current literature by adding the notion that drinking fluid according to thirst sensation is unlikely to provide any worthwhile benefit over NFI during a 1 h cycling time-trial performance. 

Contrary to our hypothesis, there was no significant difference in mean speed, the amount of distance completed or mean power output maintained during the time-trial between NFI, TDFI and PFI. That PFI offers no performance improvement over NFI during a 1 h cycling time-trial performance has also been observed before and is not new [3,4,5]. Interestingly, Robinson, Hawley, Palmer, Wilson, Gray, Noakes and Dennis [7] have even reported that PFI impairs 1 h cycling time-trial performance, compared with NFI. The authors concluded that an uncomfortable feeling of stomach fullness that persisted throughout exercise may have been the culprit. In studies using exercise protocols combining a period of fixed-intensity cycling exercise followed by a time-trial, mixed findings were observed, where on the one hand, Below, Mora-Rodriguez, Gonzalez-Alonso and Coyle [8] observed an advantage of PFI over NFI whereas, on the other, McConell, Stephens and Canny [6] observed no significant difference between NFI and PFI. However, as those studies used non-ecologically valid exercise protocols, and that under this situation the impact of dehydration has been shown to differ from that observed during ecologically valid exercise protocols [31], it is difficult to infer their finding to out-of-doors exercise. 

It was our contention that TDFI would improve performance over PFI primarily by substantially reducing the sensation of abdominal discomfort, which has been associated with performance impairments in other studies [7,11,13,32], and over NFI by attenuating the increase in plasma sodium concentration, and hence plasma osmolality, which has been suggested to be a mediator of performance through the regulation of thirst sensation [33]. Given that there was no significant difference in abdominal discomfort between PFI and TDFI, and that the sensation of discomfort was found to be low in both experiments, we are therefore not surprised that full fluid replacement did not hinder performance, compared with TDFI. Previous studies examining the impact of hydration on 1 h cycling performance have observed significant gastrointestinal discomforts in their participants [4,6,7]. Contrary to ours, these studies all provided an important bolus of fluid immediately prior to the beginning of exercise, which may potentially explain the divergence in findings. On the other hand, although thirst sensation was found to be significantly higher with NFI than TDFI, from a physiological perspective the difference between trials was likely trivial, reaching on the perception scale an average rating corresponding to moderate thirst for NFI and slight thirst for TDFI. Moreover, the magnitude of difference in plasma sodium changes among conditions was found not to be statistically different. 

When racing against the clock, individuals pace their speed and modify their behavior in effort to achieve optimal sensory sensation, and feedback provided by perceived exertion plays a pivotal role in the different decision-making processes. We observed no significant difference in perceived exertion throughout exercise among the different experiments, suggesting that the level of stress placed on the body and imposed to the different physiological systems was similar among experiments, thereby providing another potential explanation for the lack of difference in performance among trials. 

To prevent the impairment of, or reduce the decline in, exercise performance as well as for maintaining cardiovascular and thermoregulatory functions, it is proposed that the goal of drinking during exercise is to prevent a body mass loss >2% [34]. Yet, despite exceeding this body mass loss threshold during both the NFI and TDFI condition, no decline in performance was observed compared with PFI, where body mass loss was kept <2%, as currently recommended by some organizations [34,35]. Moreover, trivial differences from a physiological standpoint were observed among all three experiments for heart rate (≤6 beats · min^-1^) and rectal temperature (≤0.2 °C). Albeit it can be argued that participants did not exercise long enough with a body mass loss >2% to observe the detrimental effect of dehydration during TDFI, this argument does not hold for the NFI trial where participants exercised for the last 20 min with a body mass loss >2%. Although heart rate and rectal temperature were both elevated among all trials during exercise, the lack of difference in the regulation of these variables among trials could potentially be explained by the combination of the large core-to-skin gradient in temperature coupled with the similar regulation of plasma volume changes among trials, which together likely allowed the maintenance of similar skin blood flow and venous return to the heart despite the varying degrees of BM loss. Moreover, it must be taken into account that this study was performed in trained athletes, who show improved cardiovascular and thermoregulatory functions under an hypohydration state, compared to untrained individuals [36].

Several factors need to be considered when interpreting the present findings. First, participants entered the study with different states of heat acclimatization, as the collection of data took place from the end of the summer to the beginning of the winter months. However, considering the crossover design of the study, we believe that this factor played a marginal role. Moreover, regulatory control of physiological functions and water balance would have been even tighter in heat-acclimatized participants [37], suggesting that it is unlikely that different findings would have been observed had participants been accustomed to the heat. Although all participants trained >10 h·week^−1^, not all of them were high-level competitive cyclists. Having worked exclusively with those athletes would have likely lowered the variation in performance among trials, potentially helping to detect worthwhile changes in time-trial performance. Participants were given access to their power output, time, speed and distance so they could optimize their pacing strategy during the time-trial. This could be seen as a weakness of this study. We argue the opposite, as under real-world conditions, cyclists rely on these parameters to adjust pacing, especially during time-trial conditions. Moreover, had the impact of dehydration been so deleterious on performance, participants would likely have been unable to rely on their usual well-hydrated parameters to adjust pacing. Three high-speed fans blowing air at a speed of 25–30 km·h^−1^ were placed in front of the participants during exercise. This variable has often been neglected in many studies even if it has been well established that it plays an important role in the control of thermoregulation [38]. 

In conclusion, our results indicate that drinking according to thirst sensation during a 1 h cycling time-trial performance confers, from a statistical standpoint, no advantage compared with NFI and PFI. From a practical standpoint; however, difference in findings were found to be trivial and uncertain to determine whether PFI or TDFI offers any advantage over NFI. In a recent meta-analysis, Holland et al. [9] concluded that PFI aimed at fully replacing sweat losses impairs 1 h high-intensity cycling performance, compared with NFI. Results of the current study adds to the literature and indicate that neither PFI nor TDFI are likely to offer any advantage over NFI during a 1 h cycling time-trial.

## Figures and Tables

**Table 1:** Physical characteristics of participants (7 males and 2 females).

Characteristics	Mean ± SD
Age (years)	30 ± 9
Height (cm)	175 ± 4
Body mass (kg)	71 ± 7
Fat mass (%)	11 ± 8
Fat-free mass (%)	86 ± 8
Resting heart rate (beats·min^−1^)	60 ± 10
Maximal heart rate (beats·min^−1^)	192 ± 8
Peak oxygen consumption (mL·kg^−1^·min^−1^)	59 ± 8
Peak power output (W)	391 ± 51
Relative peak power output (W·kg^−1^)	5.5 ± 0.7

**Table 2:** Data related to the hydration state of participants before each time-trial.

Parameters	NFI	TDFI	PFI	*p*
USG (g·mL^−1^)	1.008 ± 0.007	1.008 ± 0.007	1.006 ± 0.004	0.97
Hematocrit (%)	46 ± 2	46 ± 2	45 ± 3	0.44
Body mass (kg)	72.2 ± 7.3	71.9 ± 7.4	72.1 ± 7.5	0.49
Heart rate (beats·min^−^^1^)	76 ± 12	80 ± 14	77 ± 18	0.83

Results are mean ± SD. NFI: no fluid intake; PFI: programmed fluid intake; TDFI: thirst-driven fluid intake; USG: urine specific gravity.

**Table 3:** Fluid balance data during the time-trials.

Parameters	NFI	TDFI	PFI
Total water consumption (mL)	0	565 ± 178 *	1606 ± 125 *^#^
Total water consumption (mL·kg^−1^)	0	8.0 ± 2.9 *	22.5 ± 3.3 *^#^
Total water consumption (mL·km^−1^)	0	15.8 ± 4.9 *	45.0 ± 3.7 *^#^
Sweat loss (mL)	2102 ± 159	2118 ± 129	2010 ± 191
Post-exercise USG (g·mL^−1^)	1.010 ± 0.008	1.011 ± 0.007	1.010 ± 0.005
Exercise-induced BM loss (% BM)	2.9 ± 0.4	2.2 ± 0.3 *	0.6 ± 0.2 *^#^
Rate of BM loss (% BM·min^−1^)	0.049 ± 0.006	0.036 ± 0.005 *	0.009 ± 0.004 *^#^
Time to reach 2% BM loss (min)	41.4 ± 5.1	55.9 ± 7.0 *	236.7 ± 80.3 *^#^
Post-exercise urine production (mL)	199 ± 110	152 ± 103	187 ± 145

Results are mean ± SD. * *p* < 0.01 vs. NFI, # *p* < 0.01 vs. TDFI. BM: body mass; NFI: no fluid intake; PFI: programmed fluid intake; TDFI: thirst-driven fluid intake; USG: urine specific gravity.

## References

[1] Cheuvront S.N., Kenefick R.W., Montain S.J., Sawka M.N. (2010). Mechanisms of aerobic performance impairment with heat stress and dehydration. J. Appl. Physiol..

[2] Montain S.J., Coyle E.F. (1992). Fluid ingestion during exercise increases skin blood flow independent of increases in blood volume. J. Appl. Physiol..

[3] Bachle L., Eckerson J., Albertson L., Ebersole K., Goodwin T., Petzel D. (2001). The effect of fluid replacement on endurance performance. J. Strength Cond. Res./Natl. Strength Cond. Assoc..

[4] Backx K., van Someren K.A., Palmer G.S. (2003). One hour cycling performance is not affected by ingested fluid volume. Int. J. Sport Nutr. Exerc. Metab..

[5] Kay D., Marino F.E. (2003). Failure of fluid ingestion to improve self-paced exercise performance in moderate-to-warm humid environments. J. Biol..

[6] McConell G.K., Stephens T.J., Canny B.J. (1999). Fluid ingestion does not influence intense 1-h exercise performance in a mild environment. Med. Sci. Sports Exerc..

[7] Robinson T.A., Hawley J.A., Palmer G.S., Wilson G.R., Gray D.A., Noakes T.D., Dennis S.C. (1995). Water ingestion does not improve 1-h cycling performance in moderate ambient temperatures. Eur. J. Appl. Physiol. Occup. Physiol..

[8] Below P.R., Mora-Rodriguez R., Gonzalez-Alonso J., Coyle E.F. (1995). Fluid and carbohydrate ingestion independently improve performance during 1 h of intense exercise. Med. Sci. Sports Exerc..

[9] Holland J.J., Skinner T.L., Irwin C.G., Leveritt M.D., Goulet E.D.B. (2017). The influence of drinking fluid on endurance cycling performance: A meta-analysis. Sports Med. (Auckl. N. Z.).

[10] Savoie F.A., Dion T., Asselin A., Gariepy C., Boucher P.M., Berrigan F., Goulet E.D. (2015). Intestinal temperature does not reflect rectal temperature during prolonged, intense running with cold fluid ingestion. Physiol. Meas..

[11] Daries H.N., Noakes T.D., Dennis S.C. (2000). Effect of fluid intake volume on 2-h running performances in a 25 degrees C environment. Med. Sci. Sports Exerc..

[12] Dion T., Savoie F.A., Asselin A., Gariepy C., Goulet E.D. (2013). Half-marathon running performance is not improved by a rate of fluid intake above that dictated by thirst sensation in trained distance runners. Eur. J. Appl. Physiol..

[13] Mitchell J.B., Voss K.W. (1991). The influence of volume on gastric emptying and fluid balance during prolonged exercise. Med. Sci. Sports Exerc..

[14] Goulet E.D.B., Hoffman M.D. (2019). Impact of Ad Libitum Versus Programmed Drinking on Endurance Performance: A Systematic Review with Meta-Analysis. Sports Med. (Auckl. N. Z.).

[15] Noakes T.D. (2010). Is drinking to thirst optimum?. Ann. Nutr. Metab..

[16] Dugas J.P., Oosthuizen U., Tucker R., Noakes T.D. (2009). Rates of fluid ingestion alter pacing but not thermoregulatory responses during prolonged exercise in hot and humid conditions with appropriate convective cooling. Eur. J. Appl. Physiol..

[17] Mundel T., King J., Collacott E., Jones D.A. (2006). Drink temperature influences fluid intake and endurance capacity in men during exercise in a hot, dry environment. Exp. Physiol..

[18] Lee J.K., Shirreffs S.M., Maughan R.J. (2008). Cold drink ingestion improves exercise endurance capacity in the heat. Med. Sci. Sports Exerc..

[19] Ramanathan N.L. (1964). A new weighting system for mean surface temperature of the body. J. Appl. Physiol..

[20] WHO Guidelines Approved by the Guidelines Review Committee (2010). WHO Guidelines on Drawing Blood: Best Practices in Phlebotomy.

[21] Goulet E.D.B., De La Flore A., Savoie F.A., Gosselin J. (2018). Salt + Glycerol-Induced Hyperhydration Enhances Fluid Retention More Than Salt- or Glycerol-Induced Hyperhydration. Int. J. Sport Nutr. Exerc. Metab..

[22] Dill D.B., Costill D.L. (1974). Calculation of percentage changes in volumes of blood, plasma, and red cells in dehydration. J. Appl. Physiol..

[23] Marino F.E., Kay D., Cannon J., Serwach N., Hilder M. (2002). A reproducible and variable intensity cycling performance protocol for warm conditions. J. Sci. Med. Sport.

[24] Palmer G.S., Dennis S.C., Noakes T.D., Hawley J.A. (1996). Assessment of the reproducibility of performance testing on an air-braked cycle ergometer. Int. J. Sports Med..

[25] Kruschke J.K., Liddell T.M. (2018). The Bayesian New Statistics: Hypothesis testing, estimation, meta-analysis, and power analysis from a Bayesian perspective. Psychon. Bull. Rev..

[26] Blume J.D., D’Agostino McGowan L., Dupont W.D., Greevy R.A. (2018). Second-generation p-values: Improved rigor, reproducibility, & transparency in statistical analyses. PLoS ONE.

[27] Hopkins W.G. (2004). How to interpret changes in an athletic performance test. Sportscience.

[28] Lakens D. (2013). Calculating and reporting effect sizes to facilitate cumulative science: A practical primer for t-tests and ANOVAs. Front. Psychol..

[29] Goulet E.D., Aubertin-Leheudre M., Plante G.E., Dionne I.J. (2007). A meta-analysis of the effects of glycerol-induced hyperhydration on fluid retention and endurance performance. Int. J. Sport Nutr. Exerc. Metab..

[30] Noakes T.D. (1993). Fluid replacement during exercise. Exerc. Sport Sci. Rev..

[31] Goulet E.D. (2013). Effect of exercise-induced dehydration on endurance performance: Evaluating the impact of exercise protocols on outcomes using a meta-analytic procedure. Br. J. Sports Med..

[32] Burke L.M., Wood C., Pyne D.B., Telford D.R., Saunders P.U. (2005). Effect of carbohydrate intake on half-marathon performance of well-trained runners. Int. J. Sport Nutr. Exerc. Metab..

[33] Sawka M.N., Noakes T.D. (2007). Does dehydration impair exercise performance?. Med. Sci. Sports Exerc..

[34] Sawka M.N., Burke L.M., Eichner E.R., Maughan R.J., Montain S.J., Stachenfeld N.S. (2007). American College of Sports Medicine position stand. Exercise and fluid replacement. Med. Sci. Sports Exerc..

[35] McDermott B.P., Anderson S.A., Armstrong L.E., Casa D.J., Cheuvront S.N., Cooper L., Kenney W.L., O’Connor F.G., Roberts W.O. (2017). National Athletic Trainers’ Association Position Statement: Fluid Replacement for the Physically Active. J. Athl. Train..

[36] Merry T.L., Ainslie P.N., Cotter J.D. (2010). Effects of aerobic fitness on hypohydration-induced physiological strain and exercise impairment. Acta Physiol. (Oxf. Engl.).

[37] Periard J.D., Racinais S., Sawka M.N. (2015). Adaptations and mechanisms of human heat acclimation: Applications for competitive athletes and sports. Scand. J. Med. Sci. Sports.

[38] Saunders A.G., Dugas J.P., Tucker R., Lambert M.I., Noakes T.D. (2005). The effects of different air velocities on heat storage and body temperature in humans cycling in a hot, humid environment. Acta Physiol. Scand..

